# Laparoendoscopic single-site surgery versus conventional laparoscopy for hysterectomy: a systematic review and meta-analysis

**DOI:** 10.1007/s00404-017-4323-y

**Published:** 2017-03-29

**Authors:** Evelien M. Sandberg, Claire F. la Chapelle, Marjolein M. van den Tweel, Jan W. Schoones, Frank Willem Jansen

**Affiliations:** 1 Department of Gynecology, Minimally Invasive Surgery, Leiden University Medical Center, PO Box 9600, 2300 RC Leiden, The Netherlands; 2 Walaeus Library, Leiden University Medical Center, Leiden, The Netherlands; 3 Department of Biomechanical Engineering, Delft University of Technology, Delft, The Netherlands

**Keywords:** Hysterectomy, Single-port surgery, LESS, Conventional laparoscopy

## Abstract

**Purpose:**

To assess the safety and effectiveness of LESS compared to conventional hysterectomy.

**Methods:**

The systematic review and meta-analysis was performed according to the MOOSE guideline, and quality of evidence was assessed using GRADE. Different databases were searched up to 4th of August 2016. Randomized controlled trials and cohort studies comparing LESS to the conventional laparoscopic hysterectomy were considered for inclusion.

**Results:**

Of the 668 unique articles, 23 were found relevant. We investigated safety by analyzing the complication rate and found no significant differences between both groups [OR 0.94 (0.61, 1.44), *I* ^2^ = 19%]. We assessed effectiveness by analyzing conversion risk, postoperative pain, and patient satisfaction. For conversion rates to laparotomy, no differences were identified [OR 1.60 (0.40, 6.38), *I* ^2^ = 45%]. In 3.5% of the cases in the LESS group, an additional port was needed during LESS. For postoperative pain scores and patient satisfaction, some of the included studies reported favorable results for LESS, but the clinical relevance was non-significant. Concerning secondary outcomes, only a difference in operative time was found in favor of the conventional group [MD 11.3 min (5.45–17.17), *I* ^2^ = 89%]. The quality of evidence for our primary outcomes was low or very low due to the study designs and lack of power for the specified outcomes. Therefore, caution is urged when interpreting the results.

**Conclusion:**

The single-port technique for benign hysterectomy is feasible, safe, and equally effective compared to the conventional technique. No clinically relevant advantages were identified, and as no data on cost effectiveness are available, there are currently not enough valid arguments to broadly implement LESS for hysterectomy.

**Electronic supplementary material:**

The online version of this article (doi:10.1007/s00404-017-4323-y) contains supplementary material, which is available to authorized users.

## Introduction

Since the early 1990s, “minimally invasive surgery” (MIS) has been rapidly implemented into a variety of surgical disciplines. The main advantage of minimally invasive procedures is the absence of a large abdominal wound, which results in fewer wound-related complications, less postoperative pain, and a shorter hospital stay [1]. In an effort to extend these benefits, an increasing enthusiasm has emerged for the laparoendoscopic single-site surgery (LESS). In LESS, multiple laparoscopic instruments are placed through one single abdominal incision at the place of the umbilicus. The hypothesis is that single incision technique might offer advantages over the standard multi-port laparoscopy as abdominal wall trauma is decreased, potentially leading to less postoperative pain and improved cosmesis [2–4]. The potential drawbacks of the single-port approach are a larger umbilical incision [5, 6] and the proximity of the instruments resulting in a technical challenge, especially for advanced surgery. It was only in 1991 that Pelosi et al. performed the first LESS hysterectomy [7], more than 20 years after the first publication on the LESS procedure in 1969 [6]. Reports have currently shown the feasibility of LESS surgery in many benign gynecologic procedures [8, 9]. However, it remains debatable whether this new technology has added value over the existing conventional laparoscopic technique and whether it should be broadly implemented for hysterectomy.

The proportion of laparoscopic hysterectomies (LH) has significantly increased the last decades: from 3% in 2002 to 36% in 2012 in the Netherlands [10], and similar numbers have been observed in other countries (United States [11] and Finland [12]). Regarding the proportion of hysterectomies performed using the LESS approach, no national overviews have been published on this topic so far. In some parts of the world, single-port hysterectomy seems well implemented. A retrospective single-hospital study from Korea showed for example that in 2013, 80% of their hysterectomies were LESS hysterectomies [13]. Hysterectomy in general is one of the most performed advanced surgeries in gynecology with approximately 600,000 procedures a year in the United States [11]. As a result, defining the surgical approach with the most advantages is essential. In this light, the aim of this study is to provide a systematic review and meta-analysis of the current comparative studies evaluating specifically LESS hysterectomy and conventional laparoscopy. We particularly focused on the safety and effectiveness of the two techniques.

## Materials and methods

### Eligibility criteria, information source, search strategy

This systematic review was conducted according to the MOOSE guidelines [14]. We identified original published studies through a search of Medline (PubMed version), EMBASE (Ovid version), Cochrane, Web of Science, Central, CINAHL, Academic Search Premier and Science Direct up to 4th of Augustus 2016 without restriction. The search terms included ‘gynecology’, ‘hysterectomy’, and all acronyms of LESS. The exact search terms are presented in supplemented material (Appendix 1). In addition, relevant studies cited in the reference lists of the selected papers were evaluated. Only comparative studies (randomized controlled trials, prospective and retrospective cohort studies) evaluating LESS versus hysterectomy for benign indications were considered for inclusion. LESS procedures had to be strictly performed through one single (umbilical) port as opposed to the conventional laparoscopic hysterectomy performed through more than one port. Studies on animals or patients aged <18 years were excluded as well as studies comprising endoscopic surgery with different techniques (e.g., hand- or robot-assisted, isobaric pneumoperitoneum). We also excluded descriptive review articles, surveys, technical reports, published abstracts without a full manuscript, reports from meetings, and trials with less than ten included participants per arm or 20 in total.

### Study selection

Two reviewers independently screened the titles and abstracts for their relevance (ES and CC). Potentially relevant studies were obtained in full text and assessed for inclusion. We included studies wherein the effectiveness and/or safety of LESS compared to conventional laparoscopy for hysterectomy were investigated. To assess the safety of a procedure, we considered complication rates as primary outcome. Effectiveness refers to the potential success of a surgical procedure, and therefore, we considered: success rate (defined by the chance for a successful procedure without conversion to laparotomy and for the use of an additional port in the single-site group), postoperative pain scores, cosmetic outcomes, and patient satisfaction (including sexual function) as relevant primary outcomes. The following secondary perioperative outcomes were considered: operative time, intraoperative blood loss, and length of hospital stay. Although less important, these are also relevant identifiers for the effectiveness of a procedure.

Complications were defined according to the classification of the Dutch Society of Obstetrics and Gynecology and further divided into ‘major complications’ and ‘minor complications’ [15]. Major complications included: major hemorrhage or hematoma (requiring transfusion); urinary tract or bowel injury; pulmonary embolism; major anesthesia problems; vaginal cuff dehiscence; port site hernia; and re-operation. Minor complications were defined as hemorrhage (not requiring transfusion) or hematoma (with spontaneous drainage); infection to the chest, urinary tract, wound, pelvic, other, or pyrexia 38 °C; deep vein thrombosis; and other minor complication requiring treatment (including voiding dysfunction and ileus). We distinguished two types of conversion: an unintended conversion to laparotomy and the need for an additional port in the single-site group. The postoperative pain should be expressed on a self-reported scale [e.g., visual analogous scale (VAS), numerical rating scale (NRS)] [16], and for cosmetic outcomes, validated questionnaires should be used.

### Data extraction

Outcome data as mentioned in the previous heading as well as study and patient characteristics were extracted from the included studies. These baseline findings included study design, number of included participants, country where the study was conducted, source of funding, relevant characteristics of the participants (age, body mass index, and uterine weight), description of the procedural setting, and experience of the physician. Data related to the defined outcomes were assessed for inclusion in the meta-analysis. Sensitivity analyses were performed for randomized studies and cohort studies when relevant subgroup analyses were accomplished for TLH and LAVH.

### Assessment of risk of bias

The study limitations in randomized trials and observational studies were assessed using the checklists adapted from Guyatt et al. [17]: (1) random sequence generation; (2) allocation concealment; (3) blinding of participants, surgeons, and investigators; (4) attrition bias: loss to follow-up (5) reporting bias: selective reporting and/or missing per protocol analysis; (6) other, e.g., use of non-validated outcome measures, difference in baseline characteristics between the groups and influence of co-interventions, or differing surgical experience in the compared procedures. For the first three points of the checklist, retrospective studies were rated as ‘high risk’, whereas attrition bias and reporting bias were marked as ‘unclear’, unless there was an additional reason to judge them as ‘high risk’. The quality of evidence was then rated following the Grading of Recommendations Assessment, Development, and Evaluation (GRADE) approach [18]. The quality of evidence was classified into one of four categories: high quality, moderate quality, low quality, or very low quality. We used the online GRADE program (GRADEpro Guideline Development Tool [Software], McMaster University, 2015, developed by Evidence Prime, Inc., available from gradepro.org). Any discrepancies between reviewers were addressed by an open discussion.

### Data/Evidence synthesis and statistical analysis

Meta-analysis was conducted using Review Manager (Version 5.2. Copenhagen: The Nordic Cochrane Centre, The Cochrane Collaboration, 2012). For continuous data, we calculated mean differences (MDs) and standard deviations (SDs); for dichotomous data, we calculated odds ratio (OD) with their 95% confidence intervals (CIs). When summary data were missing, e.g., only the median and range were available, data were transformed as appropriate according to the definitions described by Hozo [19]. We applied the random-effects model to combine data for meta-analysis.

## Results

### Study selection

**Fig. 1:**
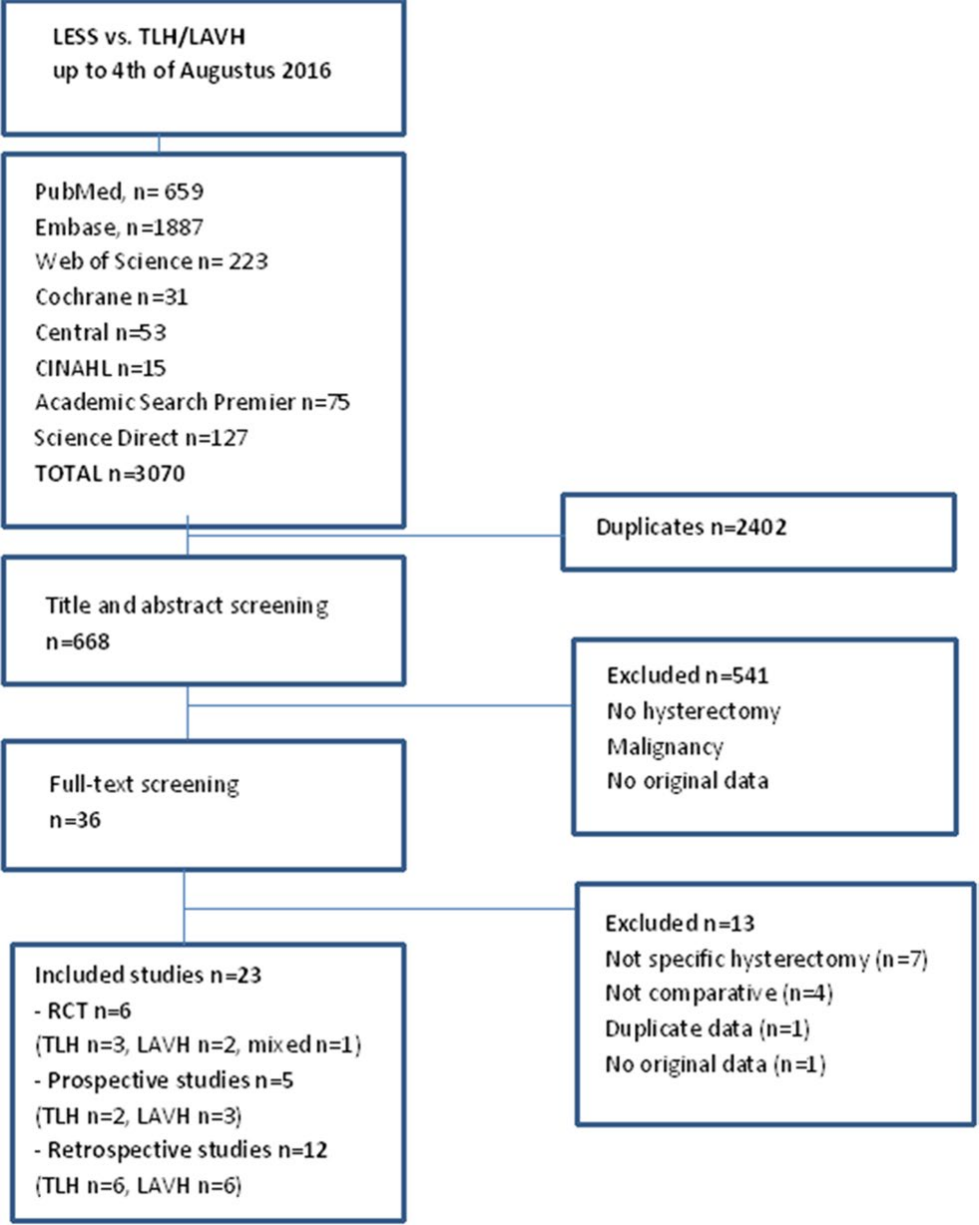
Flow diagram of the literature search

Figure 1 shows the flow diagram of the literature selection for this review. The initial search yielded 668 unique references, and twenty-three studies fulfilled our inclusion criteria. Eleven studies compared LESS hysterectomy to conventional TLH [13, 20–29], eleven studies compared LESS hysterectomy to LAVH [30–40], and in one study, both procedures were included [41]. Two studies also included supra-cervical hysterectomies [20, 21]. The study by Koyanagi [42] was excluded as all data were already included in another study by the same author [40]. The selected papers were published between 2010 and 2015.

### Study characteristics

The included studies on LESS hysterectomy versus conventional hysterectomy are described in detail in the tables ‘characteristics of included studies’ (Appendix 2). A total of 1985 women in the LESS group and 2466 women in the conventional hysterectomy were included in six randomized controlled trials [23, 24, 26, 30, 39, 41], five prospective cohort studies [21, 27, 32, 36, 37], and 12 retrospective cohort studies [13, 20, 22, 25, 28, 29, 31, 33–35, 38, 40]. Twenty of the studies (86.9%) were performed in Asia (fifteen in Korea [13, 23–25, 27, 28, 31, 32, 34–39, 41], one in China [26], two in Japan [29, 40], and two in Taiwan [30, 33]), and the other three studies originated from the United States [20], Italy [22], and France [21]. Fourteen studies had a single center design [20–24, 26–30, 33, 36, 37, 39], one RCT was multi-center, and in the other eight studies, the setting was unclear [13, 25, 31, 32, 34, 35, 38, 40].

Fifteen studies stated that there was no potential conflict of interest to disclose [13, 20–27, 30–33, 35, 38], five studies reported financial support (from a grant of Samsung Medical Center [39], from a grant of Korea Health Care technology [36, 37], from Covidien [41], and from Kyung Hee University Research Fund [34]), and three studies remained unclear about their potential conflicts [28, 29, 40].

Women in the LESS group aged between 40.3 and 53 years, their BMI ranged from 22.0 to 28.7 kg/m^2^, and their uterine weight ranged from 105 to 642 grams. In the conventional group, the age-range of the patients, their BMI, and uterine weight varied, respectively, between 41.26 and 63 years; 22.0–28.8 kg/m^2^ and 9–613 g. In two studies from Lee et al., the same cohort was partially used: the smaller cohort study focused on outcomes of sexual function. We used the data from the largest cohort [37], but for analysis of the outcome ‘sexual function’, we extracted the data from the partial cohort [36].

### Risk of bias of the included studies

**Fig. 2:**
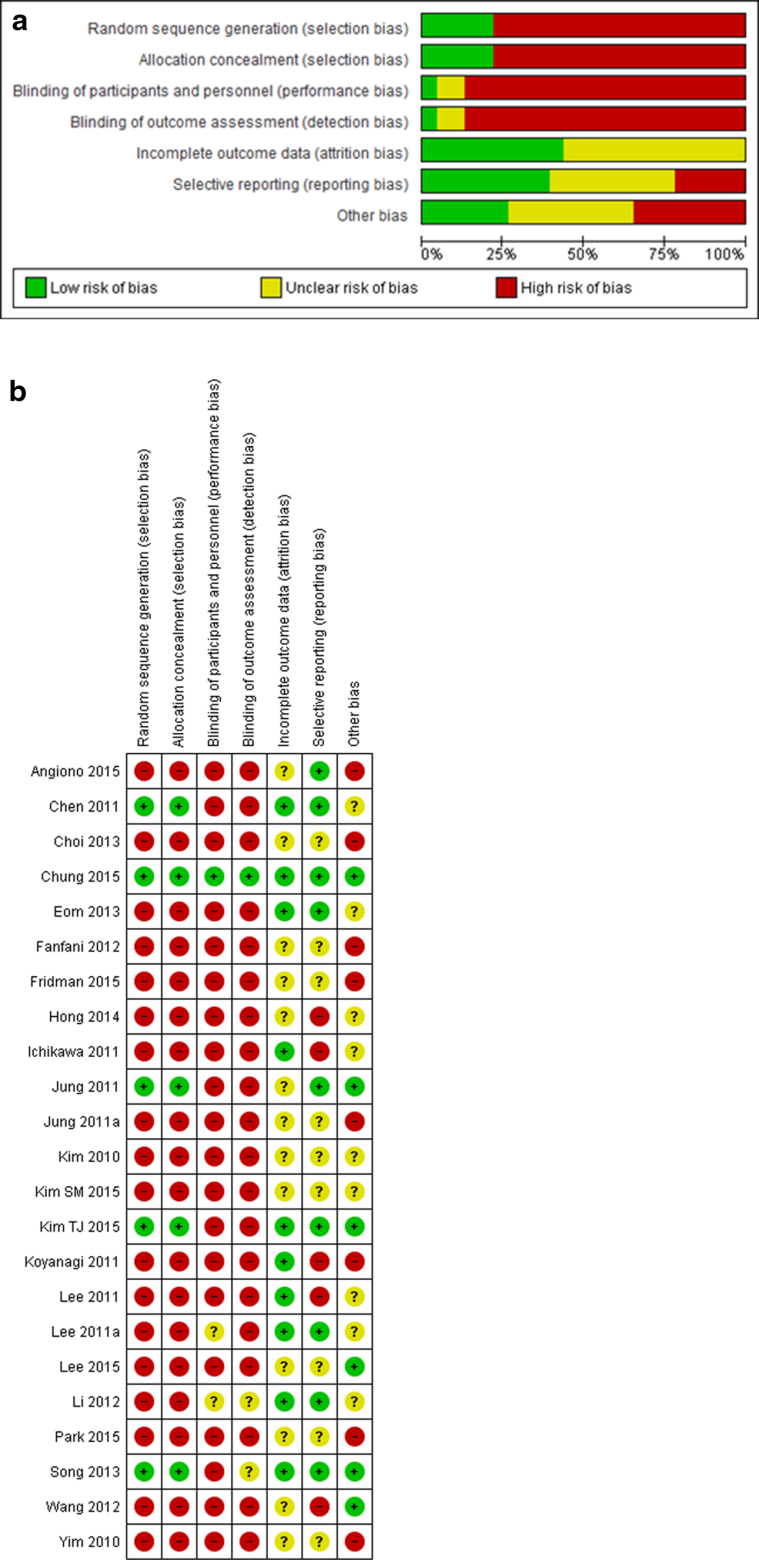
Risk of bias summary LESS versus conventional laparoscopic hysterectomy

A summary of risk of bias for the individual studies is depicted in Fig. 2. For the overview of GRADE findings, see Table 1.

**Table 1:** GRADE evidence LESS versus conventional laparoscopic hysterectomy

LESS compared to conventional for laparoscopic hysterectomy Bibliography											
Quality assessment							Summary of findings				
No of participants (studies) Follow-up	Risk of bias	Inconsistency	Indirectness	Imprecision	Publication bias	Overall quality of evidence	Study event rates (%)		Relative effect (95% CI)	Anticipated absolute effects	
							With conventional	With LESS		Risk with conventional	Risk difference with LESS
Complications MAJOR											
3943 (23 observational studies)	Serious^a^	Not serious	Not serious	Serious^b^	None	⨁◯◯◯ VERY LOW	121/2153 (5.6%)	94/1790 (5.3%)	OR 0.94 (0.61 to 1.44)	56 per 1000	3 fewer per 1000 (21 fewer to 23 more)
Complications MINOR											
2555 (13 observational studies)	Serious^a^	Not serious	Not serious	Serious^b^	None	⨁◯◯◯ VERY LOW	61/1368 (4.5%)	40/1187 (3.4%)	OR 0.76 (0.46 to 1.27)	45 per 1000	10 fewer per 1000 (24 fewer to 11 more)
Conversion to laparotomy											
4124 (21 observational studies)	Serious^a^	Not serious	Not serious	Very serious^b^	None	⨁◯◯◯ VERY LOW	8/2289 (0.3%)	22/1835 (1.2%)	OR 1.60 (0.40 to 6.38)	3 per 1000	2 more per 1000 (2 fewer to 18 more)
VAS score 24 h postoperatively											
512 (5 RCTs)	Serious^c^	Serious^d^	Not serious	Not serious	none	⨁⨁◯◯ LOW	257	255	–	The mean VAS score 24 h postoperatively was −0.15 VAS	MD 0.14 VAS lower (0.58 lower to 0.28 higher)
Cosmetic outcomes											
353 (3 RCTs)	Serious^c^	Not serious	Not serious	Serious^e,f^	None	⨁⨁◯◯ LOW	179	174	–	The mean cosmetic outcomes was 0	MD 0 (0 to 0)
Operative time											
620 (5 RCTs)	Not serious	Not serious	Not serious	Serious^g^	None	⨁⨁⨁◯ MODERATE	313	307	–	The mean operative time was 119.6 min	MD 13.14 min more (1.69 more to 24.59 more)
Blood loss											
620 (6 RCTs)	Not serious	Not serious	Not serious	Not serious	None	⨁⨁⨁⨁ HIGH	313	307	–	The mean blood loss was 158 mL	MD 5.62 mL more (0.42 more to 10.82 more)
Length of stay											
562 (4 RCTs)	Not serious	Not serious	Not serious	Not serious	None	⨁⨁⨁⨁ HIGH	284	278	–	The mean length of stay was 3.81 days	MD 0.29 days fewer (0.74 fewer to 0.17 more)

*CI* confidence interval, *OR* odds ratio, *MD* mean difference

^a^Majority of studies are retrospective cohort studies

^b^Wide confidence interval, crossing the line of no effect

^c^No blinding

^d^Differences between studies (in favor of conventional LH; in favor of LESS)

^e^Different questionnaires

^f^Underpowered

^g^For TLH versus LESS, a significant difference of 21 min was observed. For LAVH versus LESS, a non-significant difference of 2 min was observed

### Safety: complications

**Fig. 3:**
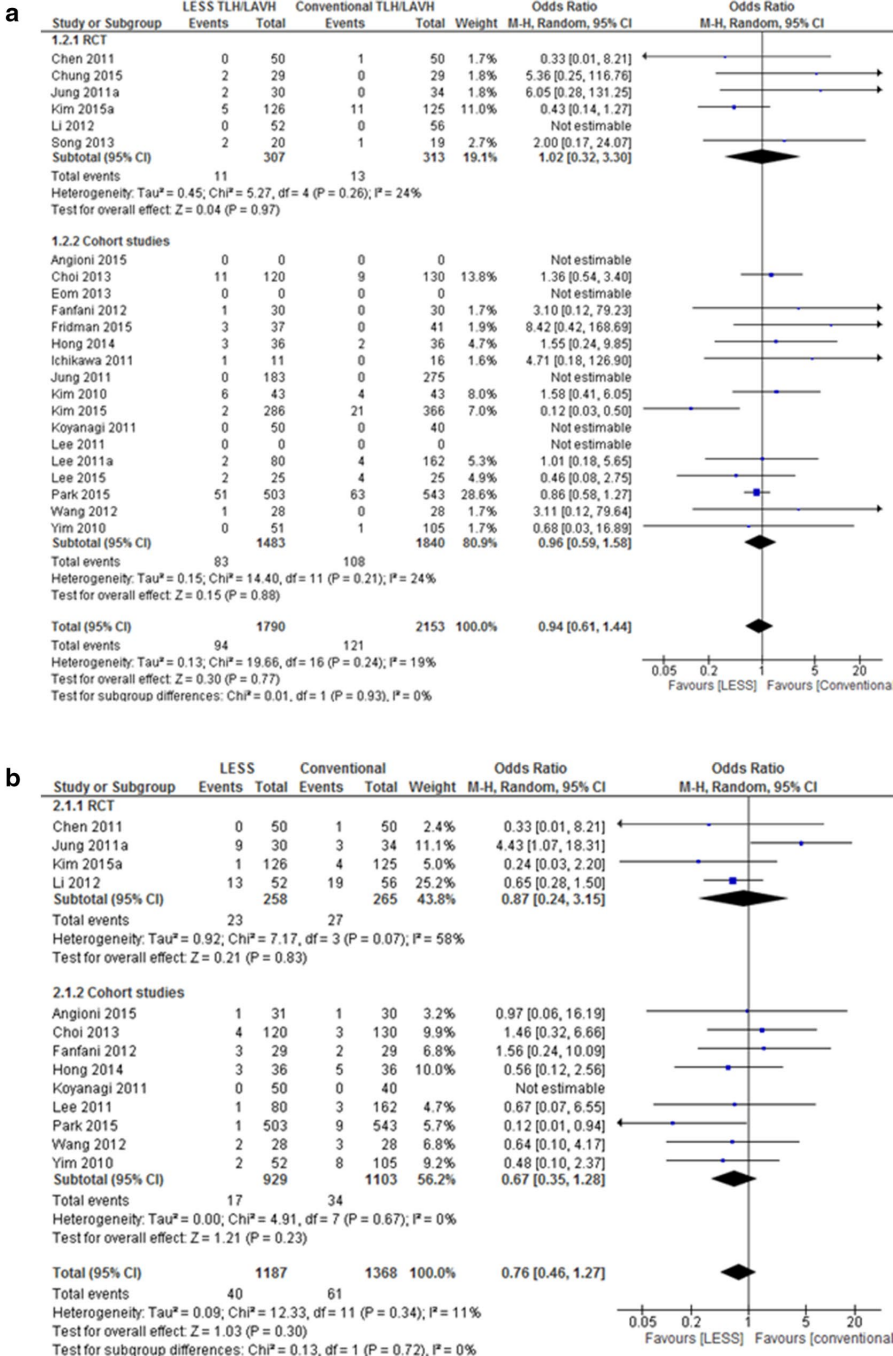

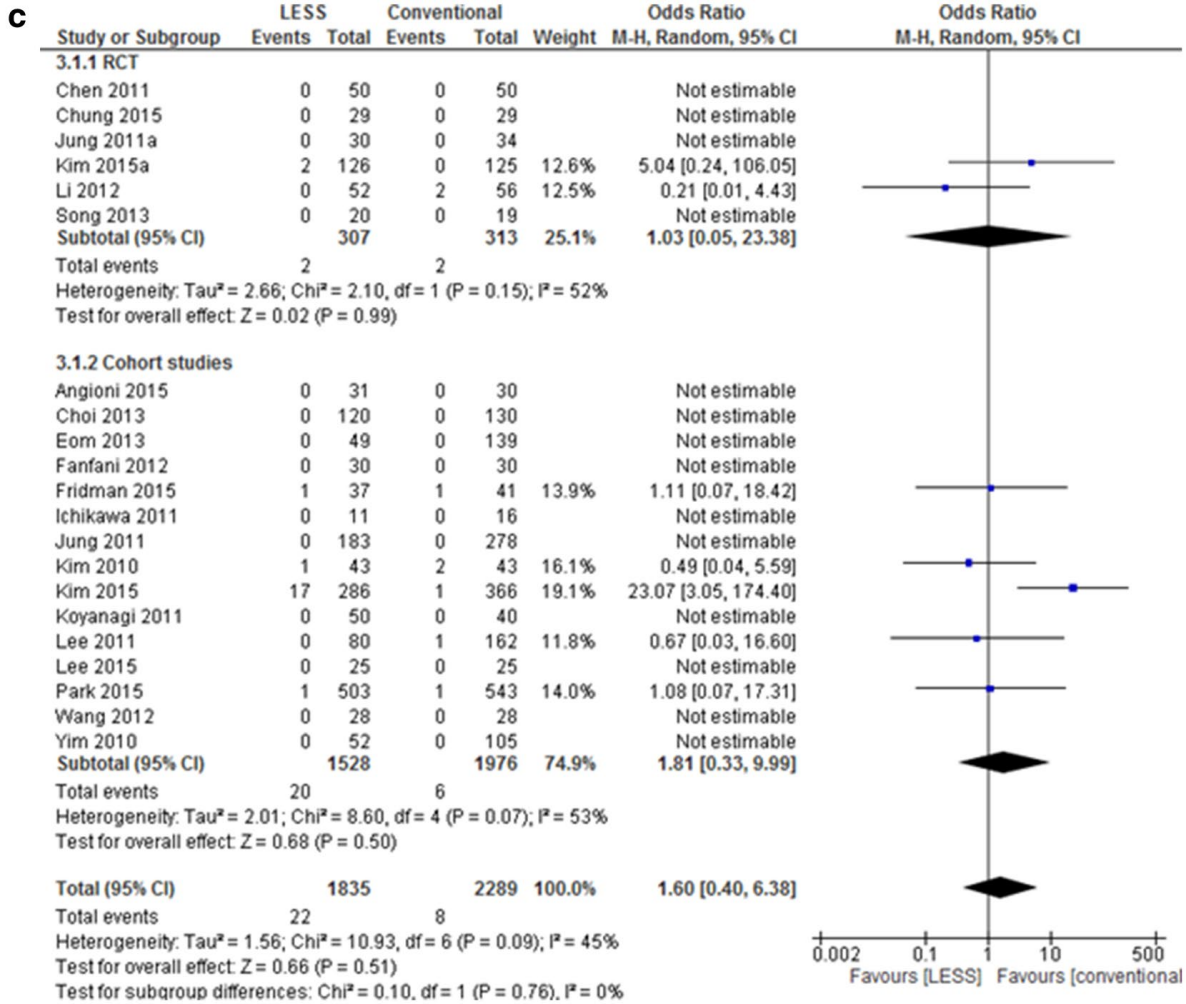
Meta-analysis of complications LESS versus conventional laparoscopic hysterectomy

We found no differences between complication rates when comparing LESS hysterectomy to conventional hysterectomy when clustering into major complications (23 studies, OR 0.94 (0.61, 1.44), *I* ^2^ = 19%, Fig. 3a) and minor complications (13 studies, OR 0.76 (0.46–1.27), *I* ^2^ = 11%, Fig. 3b). Sub-analysis specific for TLH and LAVH showed no difference (data not shown). None of the studies reported a port site herniation, though only one study mentioned that they had collected data on herniations [26].

### Effectiveness: success rate, postoperative pain scores, cosmetic results, and patient satisfaction

Conversion to laparotomy occurred in 22 of 1835 patients (1.2%) in the LESS group, compared to 8 of 2289 (0.35%) patients in the conventional group, which was not statistically significant (total 21 studies, OR 1.60 (0.40, 6.38), *I* ^2^ = 45%, Fig. 3c). The six RCTs included and reported two conversions in both groups. For the 15 cohort studies, seventeen of the 20 conversions in the LESS group were observed in one study [13]. Reason for conversions was extensive adhesions (*n* = 18), bladder injury (*n* = 1), bladder and bowel injury (*n* = 1), retroperitoneal bleeding (*n* = 1), and unspecified (*n* = 9). When evaluating the rate of additional ports needed during LESS surgery, 48 of the 1344 (3.5%) patients included had at least one additional port during LESS surgery versus one in the conventional group (0.06%) [38]. Fourteen of these cases can be attributed to Fridman et al. where additional port was needed in 38% of the cases [20]. In the study by Jung et al. one patient had an additional port due to an incidental finding of an appendiceal mucinous adenoma [34].

**Fig. 4:**
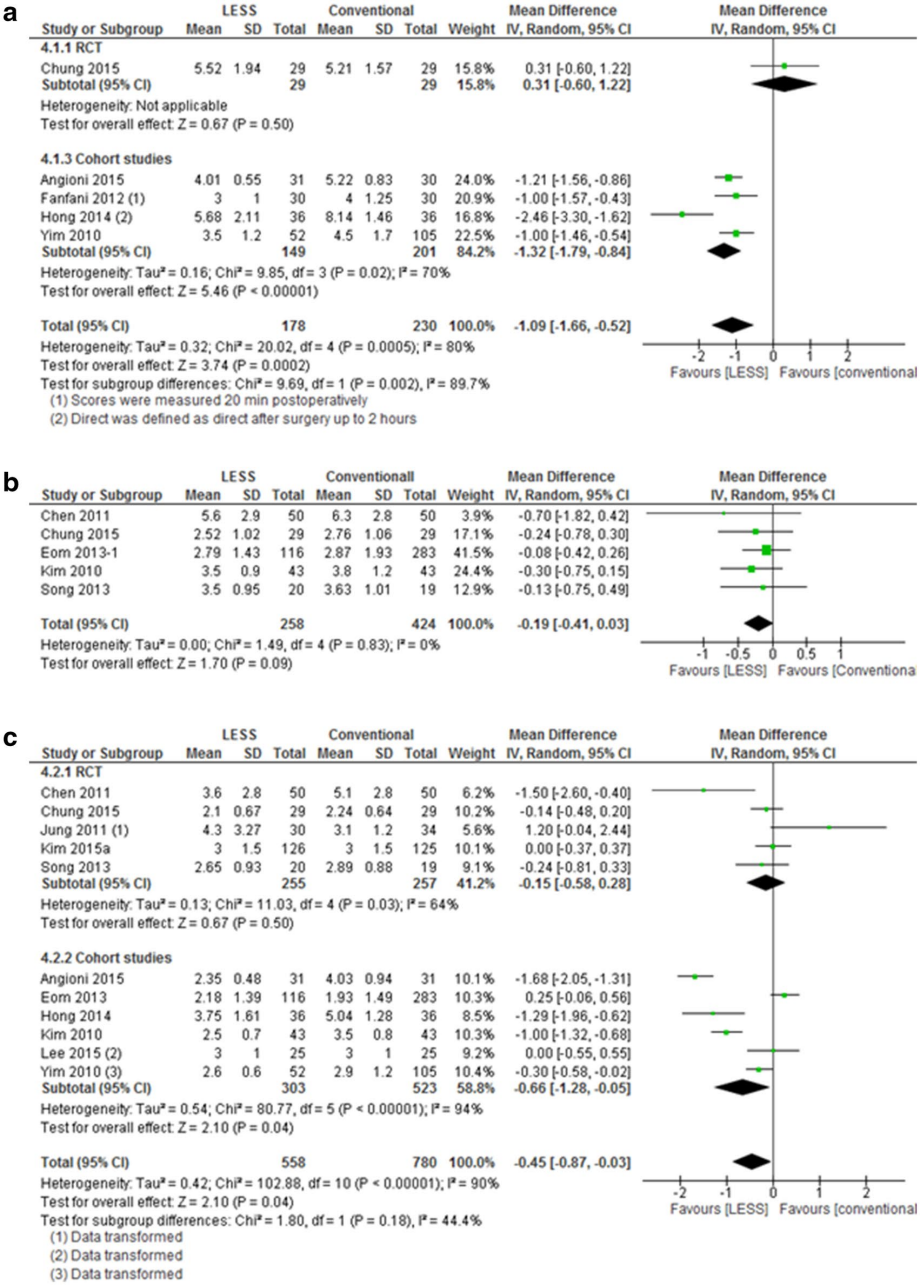
Meta-analysis of pain scores LESS versus conventional laparoscopic hysterectomy

Thirteen studies assessed the pain scores of their patients at various postoperative moments (direct after surgery up to one week) using VAS scores. Five of these studies were RCTs and one had appropriate double blinding. That specific RCT found no difference between the two groups at any of the reported moments (direct, 12, 24, and 48 h postoperative) [23]. The pain scores direct, 12 and 24 h after surgery were most frequently studied and, therefore, pooled for meta-analysis. Data that analyzed pain scores in the recovery unit, thus immediately after surgery, showed significantly lower pain scores after LESS hysterectomy compared to conventional hysterectomy (5 studies, MD −1.09 (−1.66, −0.52), *I* ^2^ = 80%, Fig. 4a) [21–23, 28]. The only randomized controlled trial included in this sub-analysis showed no difference between the two groups. At 12 h, a non-significant difference was observed (5 studies, MD −0.19 (−0.41, 0.03), *I* ^2^ = 0%, Fig. 4b). At 24 h, meta-analysis showed a significant difference between the two groups (11 studies, MD −0.45 (−0.87, −0.03), *I* ^2^ = 90%, Fig. 4c) [21, 23, 25, 28]. Though, the subgroup analysis including five RCTs showed non-significant results (MD −0.15 [−0.58, 0.28]. *I* ^2^ = 64%).

Ten studies reported on data regarding analgesic use [22–25, 28, 30, 33, 38, 39, 41]. Chung et al. and Jung et al. showed that the LESS group requested significantly more (additional) analgesics, but the VAS scores revealed no difference [23, 24]. In contrast, the (rescue) analgesic requirement was significantly lower in the LESS group in four studies [22, 28, 30, 38]. Similarly, Hong et al. calculated a pain-relief score based on the amount and type of analgesic used and the effectiveness on pain relief and their results were also in favor of the single-port surgery [33]. Finally, Lee et al. [25], Kim et al. 41], and Song et al. [39] showed no difference in analgesic use between the two groups.

Three studies reported on cosmetic results [21, 39, 41], and two used the validated Body Image Questionnaire at 1, 4, and 24 week postoperative. Patients in the LESS group were significantly more satisfied with their scars and had higher satisfaction with their own body at the three measured moments. Kim et al. studied the scar satisfaction using the patient and observer scar assessment scale (POSAS) 1 week and 2 months after surgery and showed no difference between the single-site group and the multi-port one. Li et al. studied patient satisfaction and demonstrated a higher patient satisfaction rate in the single-port group, although it was unclear which questionnaire was used [26]. Lee et al. compared the sexual function of premenopausal women by using the female sexual function index and showed no difference between women that underwent LESS compared to LAVH [36].

### Secondary outcomes

**Fig. 5:**
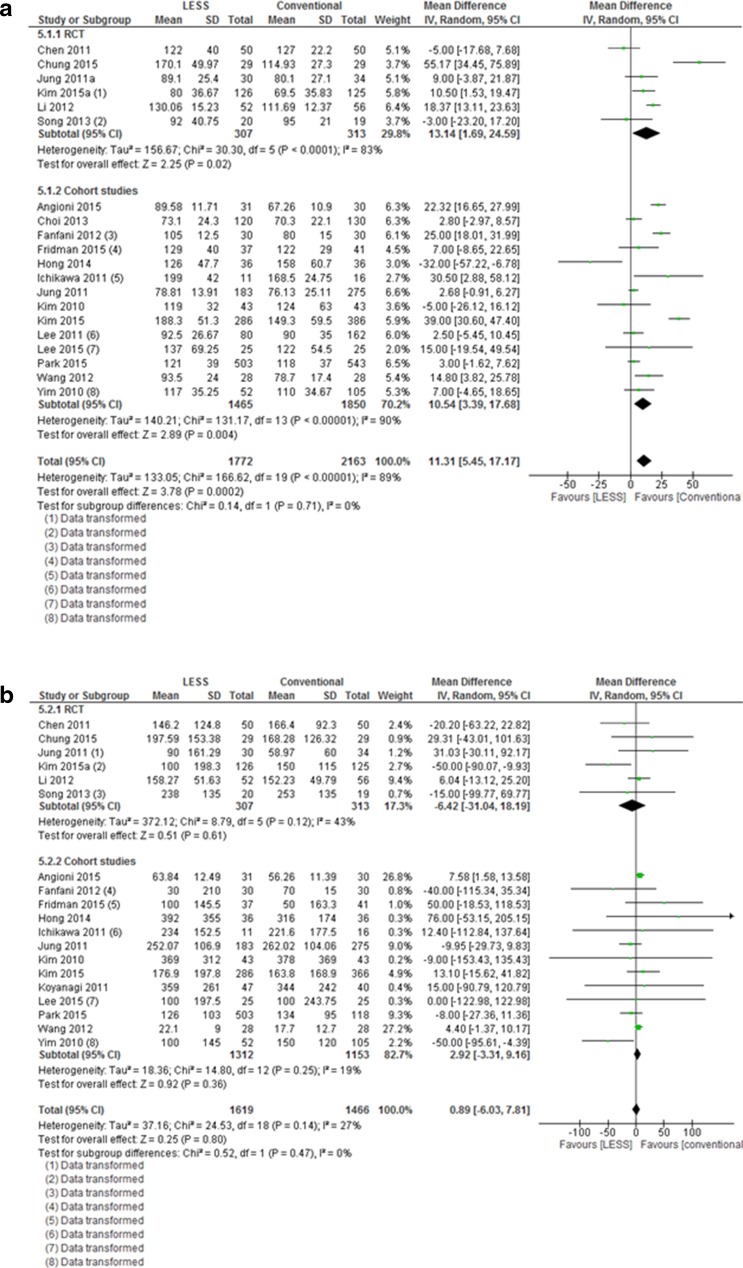

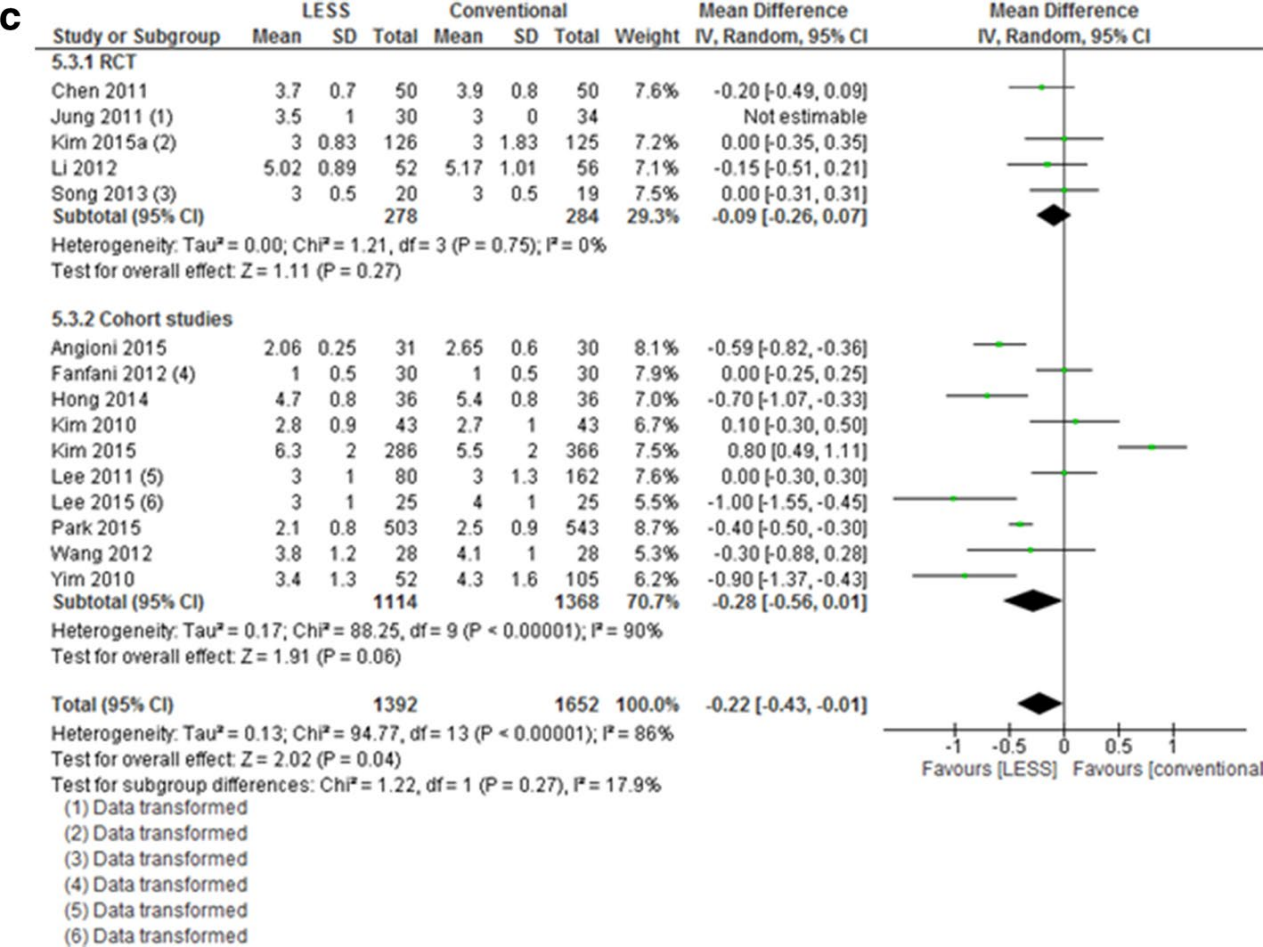
Meta-analysis of surgical outcomes from LESS versus conventional laparoscopic hysterectomy (operative time, blood loss, and length of stay)

The operative time was significantly longer in the single-port group compared to the multi-port group (20 studies, MD 11.3 min (5.45–17.17), *I* ^2^ = 89%, Fig. 5a). When comparing separately TLH and LAVH, a significant difference of 21 min was seen in favor of the TLH group, compared to a non-significant difference of 2 min after LAVH (data not shown). No difference was seen for the intraoperative blood loss (19 studies, MD 1 mL (−6.03, −7.81),* I* ^2^ =27%, Fig. 5b). For the length of hospital stay, a small significant difference was seen (15 studies, MD −0.22 (−0.43, −0.01), *I* ^2^ = 86%, Fig. 5c). This difference was not seen when looking separately at the RCTs and cohort studies.

## Discussion

### Main findings

In this systematic review, we evaluated the safety and effectiveness of LESS hysterectomy compared to the conventional laparoscopic hysterectomy (TLH and LAVH). Twenty-three studies on LESS versus conventional hysterectomy showed no differences for safety with very low quality evidence. Concerning effectiveness, very low quality evidence indicated no difference for the risk of conversion to laparotomy in the LESS group compared to TLH and LAVH. In 3.5%, the LESS approach failed as an additional port was needed. For postoperative pain, low quality of evidence indicated a lower VAS score of 1.09 and 0.45, respectively, directly and 24 h after LESS hysterectomy, though with substantial statistical heterogeneity. Two out of three studies with low-quality evidence indicated a better cosmetic outcome after LESS versus conventional hysterectomy. A major shortcoming in these studies is the lack of a pre-operative assessment. Without a pre-operative assessment, it remains unclear whether there were any differences between the groups prior to their surgery. The third study, an RCT showed no difference with respect to scar satisfaction.

### Strengths and limitations

Though there are some RCTs available comparing LESS to conventional hysterectomy, we decided to include other comparative study designs as well. The inclusion of non-RCT designs results in less homogenous groups, but when outcomes of interest are infrequent (e.g., conversion-to-laparotomy risk, complication risks); RCTs are rarely large and lengthy enough to measure infrequent outcomes accurately. Cohort studies facilitate a larger study population and adequate power to identify significant differences. Therefore, the inclusion of study designs other than RCTs can be seen as a limitation but also as strength. In addition, to limit bias, we performed sensitivity analysis for the study design for the meta-analysis. Another strength of this review is the assessment of the quality of evidence using GRADE methodology. We believe that the use of GRADE results in additional clinical value of this review: GRADE optimizes the presentation of evidence for clinical practice. The results of this systematic review are strengthened through the findings of other reviews published on the subject that as well found no significant difference in the frequency of perioperative complications and postoperative pain scores [8, 9, 43]. Though, other reviews described a higher rate of ‘failures’ in the LESS group. These studies defined ‘failure’ as the need to convert to laparotomy and/or to add an extra port, without differentiating. We found that in 3.5% of the LESS procedures, an additional port was needed compared to < 1% in the conventional procedures.

### Interpretation

The feasibility of LESS surgery for benign gynecologic procedures seems proven [8, 9]. The meta-analyses in this review showed no significant differences in complication and conversion-rate to laparotomy between LESS and conventional hysterectomy. Without substantial statistical heterogeneity, we consider these findings reliable. Besides complication risk, the pain experienced after surgery is an important consideration and usually an important argument in favor of LESS. Though, we did not find any clinically significant differences in postoperative pain. Directly and 24 h after LESS hysterectomy, a significant lower VAS score was observed. This difference was not observed when analyzing only the RCTs. Furthermore, the mean difference did not exceed 1.09 and studies have shown that a mean difference of 2 points on a 10-point scale should be considered as clinically relevant [44]. In addition, it cannot be excluded that enrolled patients in the study are biased with respect to their pain outcomes as, except in one study, the included patients were not blinded to the type of surgery. One single randomized controlled trial applied accurate blinding [23]: patients and anesthesiology staff who measured the postoperative pain scores did not know which type of approached had been performed and similar pain scores were found. Cosmetic outcomes are also suggested as important improvement in the single-site approach but surprisingly few studies on LESS hysterectomy reported on this topic [21, 39, 41]. We judged the assessment in the two studies on patient satisfaction insufficient, since baseline assessment of body image and cosmetic satisfaction was not performed. The largest RCT published so far for hysterectomy reported no significant differences regarding scar satisfaction between the LESS and ‘conventional’ hysterectomy group. When looking at studies published in other fields than benign gynecology, inconsistent results are found for the self-scar rating in patients who underwent LESS or conventional laparoscopic surgery [45–47]. In Tuschy et al. patients who underwent conventional gynecological laparoscopy were asked which scar they would prefer to eliminate, and for most patients, it was the umbilical one [48]. In the study by Bush et al. patients were asked their aesthetic preference regarding scars, and no differences were observed between the single-site and conventional incisions [6]. In LESS surgery, higher forces are applied on the umbilical port during tissue handling and irreversible umbilical deformation has been described [29]. It is also suggested that LESS would lead to a higher risk of port herniation as the opening of the umbilical port is larger [49–51]. Though, this could not be confirmed in the current literature, as within the short study follow-up, only one case of port herniation was reported [31].

Evaluating the secondary surgical outcomes, a notable finding is the increased operative time found in the LESS versus conventional hysterectomy group: an overall mean difference of 11 min was observed, though with substantial heterogeneity. For the TLH, the mean difference was 21 min, whereas for the LAVH, a non-significant difference of 2 min was observed. The reason for the prolonged operative time during TLH is most probably related to the difference in surgical experience. For the LAVH, it makes sense that the operative time was similar as a large part of the LESS and conventional procedure is performed vaginally, thus using exactly similar techniques. It is well known that LESS surgery is technically more challenging [8, 9, 43] and studies reporting on the learning curve in LESS have suggested that sufficient skills are acquired after 10 to 15 [3] up to 40 cases [52], especially when surgeons are already well-trained in laparoscopy. In five studies included in this review, the surgical experience of the surgeons was not described [13, 28, 30, 35, 38]. In the other included studies, the experience of surgeons was defined by terms, such as ‘very experienced’, ‘senior surgeon’, or by the number of laparoscopic and/or LESS surgeries performed in one’s career. Hence, it is difficult to interpret the impact of the skills on the outcomes. It is noteworthy mentioning that we found substantial differences in baseline characteristics between compared groups in the non-randomized studies (uterine weight [20, 21, 28], age [20], BMI [31], previous surgeries, and co-morbidities [28, 38]). This could be explained by the surgeon’s specific selection when performing a new technique in a non-randomized setting. Yet, an increased uterine weight, a high BMI, and/or previous surgical interventions are known to directly influence surgical outcomes [53] and this could lead to an overestimation of effectiveness, safety, and secondary outcomes (e.g., operative time, blood loss) for LESS outcomes. In addition, it should also be taken into account that 20 of the 23 studies originated from Asian, and therefore, the impact of Asian demographics should not be underestimated.

Remarkably, none of the included studies has taken the costs of the surgery into account, and currently, it is unknown if the LESS approach is cost effective. Despite the lack of data for LESS versus conventional hysterectomy, it can be reasoned that implementing the LESS technique in a hospital is costly as the conventional instruments do not fit and new instruments need to be purchased.

As seen with previous devices and or techniques [54], implementing new technologies in the medical field is a challenge. In contrast to the introduction of new drugs, the latest techniques and devices are usually implemented in clinical practice without proper systematic evaluation regarding their safety, effectiveness, costs, and benefits. Advantages and disadvantages only become clear with the passage of time and after the implantation phase. Considering this, it is complex to answer the question whether the single-port surgery should be an additional possibility for the minimally invasive surgery. Most of studies in the review were single center and from the same region in the world, where a lot of experienced has already been acquired with the LESS technique. Despite the amount of experience with LESS in these centers, there is still no clear added value.

In conclusion, current evidence shows that the single-port technique for benign hysterectomy (TLH and LAVH) is feasible, safe, and equally effective compared to the conventional technique. Caution is urged when interpreting the results of studies on LESS because the evidence is of low-to-very low quality. Potential benefits are sought in patient satisfaction, cosmetic satisfaction, and postoperative pain, but the small differences for these outcomes appear not to be of clinical relevance. Furthermore, surgeons and patients should be aware that in up to 3.5% of LESS hysterectomies an additional port is required resulting in failure of the “single site” approach and affecting the less invasive purpose. As no clinically relevant advantages were identified, and no data on cost effectiveness were available, there are currently no solid arguments to implement the single-port technique worldwide.

## Electronic supplementary material

Below is the link to the electronic supplementary material.

Supplementary material 1 (DOC 37 KB)

Supplementary material 2 (DOCX 92 KB)
