# Supplementary material for: Laparoendoscopic single-site surgery versus conventional laparoscopy for hysterectomy: a systematic review and meta-analysis
Source: Arch Gynecol Obstet. 2017 Mar 29;295(5):1089–103. doi: 10.1007/s00404-017-4323-y (PMC5388711; doi:10.1007/s00404-017-4323-y)
Supplement: Supplementary file 1 — Supplementary material 1 (DOC 37 KB) [file 404_2017_4323_MOESM1_ESM.doc]

**Appendix 1 --** *Search up to 4th of Augustus*

**PubMed**:

("gynaecology"[All Fields] OR "gynecology"[MeSH Terms] OR "gynecology"[All Fields] OR gynaecologic[All Fields] OR gynecologic[All Fields] OR "Genital Diseases, Female"[Mesh] OR "female genital disease"[all fields] OR "female genital diseases"[all fields] OR "Gynecologic Surgical Procedures"[Mesh]) AND (("Single Incision Laparoscopic Surgery"[All Fields] OR "laparo-endoscopic single-site surgery"[all fields] OR "One port umbilical surgery"[all fields] OR "Natural orifice transluminal endoscopic surgery"[all fields] OR "Single-incision minimally invasive surgery"[all fields] OR "Single laparoscopic incision transabdominal surgery"[all fields] OR "Single-port access"[all fields] OR "Single-port laparoscopy"[all fields] OR "Single-port incisionless conventional equipment-utilizing surgery"[all fields] OR "Umbilical laparoendoscopic single-site surgery"[all fields]) OR (("laparoscopy"[MeSH Terms] OR "laparoscopy"[All Fields] OR "laparoscopic"[All Fields] OR laparoendoscopic[All Fields]) AND (("single"[All Fields] AND (site[All Fields] OR port[all fields] OR incision[all fields] OR umbilical[all fields] OR transumbilical[all fields])) OR (single-port[all fields] OR single-site[all fields] OR single-incision[all fields])) AND ("surgery"[Subheading] OR "surgery"[All Fields] OR "surgical procedures, operative"[MeSH Terms]))) AND ("2012/05/01"[PDAT] : "3000/12/31"[PDAT])

**Embase**:

(gynaecolog*.mp. OR gynecolog*.mp. OR exp gynecology/ OR exp gynecologic disease/ OR female genital disease*.mp. OR exp gynecologic surgery/) AND (("Single Incision Laparoscopic Surgery".mp. OR "laparo-endoscopic single-site surgery".mp. OR "One port umbilical surgery".mp. OR "Natural orifice transluminal endoscopic surgery".mp. OR "Single-incision minimally invasive surgery".mp. OR "Single laparoscopic incision transabdominal surgery".mp. OR "Single-port access".mp. OR "Single-port laparoscopy".mp. OR "Single-port incisionless conventional equipment-utilizing surgery".mp. OR "Umbilical laparoendoscopic single-site surgery".mp.) OR ((exp laparoscopy/ OR exp laparoscopic surgery/ OR "laparoscopy".mp. OR "laparoscopic".mp. OR laparoendoscopic.mp.) AND (("single".mp. AND (site.mp. OR port.mp. OR incision.mp. OR umbilical.mp. OR transumbilical.mp.)) OR (single-port.mp. OR single-site.mp. OR single-incision.mp.)) AND (exp surgical technique/ OR surgery.mp. OR surgical.mp.))) AND (201236 OR 201237 OR 201238 OR 201239 OR 20124* OR 20125* OR 2013* OR 2014* OR 2015* OR 2016*).ew
