# Supplementary material for: Laparoendoscopic single-site surgery versus conventional laparoscopy for hysterectomy: a systematic review and meta-analysis
Source: Arch Gynecol Obstet. 2017 Mar 29;295(5):1089–103. doi: 10.1007/s00404-017-4323-y (PMC5388711; doi:10.1007/s00404-017-4323-y)
Supplement: Supplementary file 2 — Supplementary material 2 (DOCX 92 KB) [file 404_2017_4323_MOESM2_ESM.docx]

**Characteristics of studies LESS vs. TLH/LAVH**

(Template of Review Manager v.5.1 software designed for composing Cochrane reviews)

***Angiono 2015***

| **Methods** | Prospective case control study (June 2011 and March 2014) |
| --- | --- |
| **Participants** | 61 patients undergoing a laparoscopic supracervical hysterectomy (SH) with or without bilateral salpingo-oophorectomy.  **Setting:** University hospital, cases performed by two surgeons (both of whom had optimal laparoscopic experience and at least a year of training of single port procedures). Patients were alternatively assigned to one of the procedures  **Inclusion criteria:** no evidence of gynaecologic malignancy, normal cervical cytology, appropriate medical status for laparoscopic surgery (ASA 1-2), and a uterus < 16 weeks  **Exclusion criteria:** anesthetic contraindications for laparoscopic surgery, a uterine size over 16 gestational weeks al pelvic examination, a diagnosis or suspicion of gynecologic cancer and/or deep infiltrating endometriosis, a history of pelvic radiation therapy, more than three prior laparotomies, or an inhability to understand and provide written informed consent.  **Patient characteristics:**  Age SPAL-SH 47.9 vs MPAL-SH 49, p=NS  BMI SPAL-SH 23.89 vs. MPAL-SH 22.2, p=NS  Uterine weight: SPAL-SH 259.16 vs. MPAL-SH 296.77, p=.006 |
| **Interventions** | Single port SH (SPAL-SH) (n=31) vs. Multiport LSH (MPAL-SH) (n=30) |
| **Outcomes** | Surgical outcomes (operative time, blood loss, hospital stay, peri-operative complications)  Postoperative pain score - direct, 6 hours, 24 hours and 48 hours  Patient satisfaction (including cosmetic outcomes) - BIQ questionnaire at 1, 4 and 24 weeks |
| **Notes** | France - JMIG 2015 |

Risk of bias table

| **Bias** | **Authors' judgement** | **Support for judgement** |
| --- | --- | --- |
| Random sequence generation (selection bias) | High risk | Alternatively assigned to undergo SPLA-SH or MPAL-SH |
| Allocation concealment (selection bias) | High risk | Allocation sequence: patients were alternatively assigned and therefore intervention could be foreseen in advance. |
| Blinding of participants and personnel (performance bias) | High risk | Not reported |
| Blinding of outcome assessment (detection bias) | High risk | Not reported |
| Incomplete outcome data (attrition bias) | Unclear risk | Unclear how many patients completed the questionnaires postoperatively |
| Selective reporting (reporting bias) | Low risk |  |
| Other bias | High risk | Uterine weight significant different between the two groups. Data on surgical experience reported. |

***Chung 2015***

| **Methods** | Randomized controlled trial (March 2014 - January 2015) |
| --- | --- |
| **Participants** | 60 patients undergoing laparoscopic hysterectomy for benign gynecologic diseases  **Setting:** Large teaching hospital in Korea. Procedures were performed by one of the three experienced surgeons.  **Inclusion criteria:** Laparoscopic hysterectomy for benign gynecologic diseases; 18 years or older; in an appropriate medical status for laparoscopic surgery (ASA 1-3)  **Exclusion criteria:** Suspicion of malignancy (including also microinvasive cervical cancer), the need for simultaneous interventions such as a prolapse repair, a uterine size greater than 18 weeks of gestation, ongoing peritoneal dialysis, and any disease associated with abdominal pain such as pancreatitis.  **Patient characteristics:**  Age: SPA-TLH: 47.45 vs. MPA-TLH 47.03  BMI: SPA-TLH: 24.74 vs. MPA-TLH 24.07  Previous surgeries: SPA-TLH 5 (17.2%) vs MPA-TLH 12 (41.4%)  Uterus weight: SPA-TLH: 307.34 vs. MPA-TLH 384.66, p=.398  All patients received the same mode of anesthesia (IV-PCA, with 50 mg Tridol intravenously when VAS >5). |
| **Interventions** | SPA-TLH (n=29) vs. MPA-TLH (n=29) |
| **Outcomes** | Postoperative pain management (VAS and analgesic dose)  Surgical outcomes (including blood loss, operative time, operative complications) |
| **Notes** | Korea - ACTA Obstetricia et Gynecologica Scandinavica 2015 |

Risk of bias table

| **Bias** | **Authors' judgement** | **Support for judgement** |
| --- | --- | --- |
| Random sequence generation (selection bias) | Low risk |  |
| Allocation concealment (selection bias) | Low risk |  |
| Blinding of participants and personnel (performance bias) | Low risk | Patients and anesthesiology staff were for type of surgery |
| Blinding of outcome assessment (detection bias) | Low risk | Three skin plasters were applied to the locations of the three ports used in multi-port surgery, even if there was only a single port. |
| Incomplete outcome data (attrition bias) | Low risk |  |
| Selective reporting (reporting bias) | Low risk |  |
| Other bias | Low risk |  |

***Fanfani 2012***

| **Methods** | Single-institutional matched retrospective cohort study (May 2010 - March 2011) |
| --- | --- |
| **Participants** | 85 consecutive women undergoing total laparoscopic hysterectomy  **Setting:** single center study (type of study not described). Procedures performed by experienced laparoscopic surgeons (not further described)  **Inclusion:** no absolute contraindications to laparoscopy; uterine size < 12 weeks of pregnancy; no previous longitudinal major abdominal surgery; and informed consent.  **Exclusion:** Not defined  **Patient characteristics:**  Age: S-LPS 63 vs LESS 53 vs M-LPS 51, p=.001  BMI: S-LPS 23 vs LESS 25 vs M-LPS 24, p=.378  Uterine weight: S-LPS 110 vs. LESS 105 vs. M-LPS 98, p=.247  All patients were managed with the same standardized anesthetic protocol and postoperative analgesic therapy (Paracetamol 1000 mg) was administered only on patient's demand. |
| **Interventions** | Standard laparoscopy (S-LPS) (n=30) vs. Laparoendoscopic single-site (LESS) (n=30) vs. Mini-laparoscopy (M-LPS) (n=25) |
| **Outcomes** | Operative time  Intraoperative complications  Postoperative pain assessment (immediate postoperative period and 7-14 days postoperatively) |
| **Notes** | Italy - Surg Endosc 2012 |

Risk of bias table

| **Bias** | **Authors' judgement** | **Support for judgement** |
| --- | --- | --- |
| Random sequence generation (selection bias) | High risk | Retrospective study |
| Allocation concealment (selection bias) | High risk | Retrospective study |
| Blinding of participants and personnel (performance bias) | High risk | Retrospective study |
| Blinding of outcome assessment (detection bias) | High risk | Retrospective study |
| Incomplete outcome data (attrition bias) | Unclear risk | Retrospective study |
| Selective reporting (reporting bias) | Unclear risk | Retrospective study |
| Other bias | High risk | Significant difference with respect to age |

***Fridman 2015***

| **Methods** | Retrospective cohort study (October 2008 - February 2011) |
| --- | --- |
| **Participants** | 78 patients undergoing laparoscopic hysterectomy.  **Setting:** single-surgeon cohort (no information on experience, expect that it is a 'senior surgeon')  **Inclusion:** all women who had laparoscopic hysterectomy performed by the specific surgeon  **Exclusion:** cases that have been operated on during a 4-month period when the technique for LESS supracervical hysterectomy.  **Patient characteristics:**  Age: LESS 46.1 vs. C-LSH/TLH 44.9, p=.354  BMI: LESS 28.7 vs. C-LSH/TLH 28.8, p=.937  Uterine weight: LESS 161 gram vs. C-LSH/TLH 99 gram, p<.001 |
| **Interventions** | LESS (n=37) vs. Conventional laparoscopic hysterectomy (C-LSH/TLH) (n=41) |
| **Outcomes** | **Primary outcomes:**  Operative time  **Secondary outcomes:**  Estimated blood loss  Number of conversions  Surgical complications  Utilization of accessory ports during LESS |
| **Notes** | USA - Journal of Gynecologic Surgery 2015 |

Risk of bias table

| **Bias** | **Authors' judgement** | **Support for judgement** |
| --- | --- | --- |
| Random sequence generation (selection bias) | High risk | Retrospective study |
| Allocation concealment (selection bias) | High risk | Retrospective study |
| Blinding of participants and personnel (performance bias) | High risk | Retrospective study |
| Blinding of outcome assessment (detection bias) | High risk | Retrospective study |
| Incomplete outcome data (attrition bias) | Unclear risk | Retrospective study |
| Selective reporting (reporting bias) | Unclear risk | Cases that have been operated on during a 4-month period when the technique for LESS supracervical hysterectomy were excluded, but these four first cases were published in another paper - no serious adverse events. |
| Other bias | High risk | Significant difference in uterine weight. No data on surgical experience |

***Ichikawa 2011***

| **Methods** | Retrospective study (februari 2009-april 2011) |
| --- | --- |
| **Participants** | 27 women undergoing total laparoscopic hysterectomy.  **Setting:** single-center, no information on surgeons  **Inclusion:** unclear  **Exclusion:** unclear  **Patient characteristics:**  Age: LESS 43.7 vs. C-TLH 42.4 p=.393  BMI: LESS 22.7 vs. C-TLH 22.0 p=.309  Uterine weight: LESS 276 gram vs. C-TLH 285 gram, p=0.427 |
| **Interventions** | LESS (n=11) vs. Conventional TLH (n=16) |
| **Outcomes** | Surgical outcomes |
| **Notes** | Japan - J Nippon Med School 2011 |

Risk of bias table

| **Bias** | **Authors' judgement** | **Support for judgement** |
| --- | --- | --- |
| Random sequence generation (selection bias) | High risk |  |
| Allocation concealment (selection bias) | High risk |  |
| Blinding of participants and personnel (performance bias) | High risk |  |
| Blinding of outcome assessment (detection bias) | High risk |  |
| Incomplete outcome data (attrition bias) | Low risk |  |
| Selective reporting (reporting bias) | High risk |  |
| Other bias | Unclear risk | No data on experience  Groups similar |

***Jung 2011***

| **Methods** | Randomized controlled trial (October 2009 to March 2010) |
| --- | --- |
| **Participants** | 68 patients who underwent total laparoscopic hysterectomy  **Setting:** A tertiary teaching and research institution, all procedures by three skilled surgeons (more than 100 conventional TLH and 30 LESS cases) with the assistance of a gynecologic oncology floow and a chief resident.  **Inclusion:** Women who had an indication for hysterectomy; 20 years or older; no evidence of gynecologic malignancy on imaging studies, normal cervical cytology or histologically proven preinvasive cervical neoplasia, CA-125 of 150 IU/ml or less, appropriate medical status for laparoscopic surgery (ASA I or II), and adequate uterine size for vaginal removal (smaller than 12 gestational weeks size by pelevic examination).  **Exclusion:** History of pelvic radiation therapy, suspicion of gynecologic cancer, and more than three prior laparotomies, treatment for gastrointestinal or gynecologic malignancies.  **Patient characteristics:**  Age: LESS 48 vs. C-TLH 48.1, p=.922  BMI: LESS 23.4 vs. C-TLH 23.5, p=.846  Uterine weight: LESS 173.5 vs. C-TLH 210.9, p=.185  The intra and postoperative pain management was performed according to a standard protocol.  Post-operatively patients received Ketorolac 30mg IV; Pethidine 25 mg IM; Taniflumate 370 mg oral. |
| **Interventions** | LESS (n=30) vs. conventional TLH (C-TLH) (n=34) |
| **Outcomes** | **Primary outcome:**  Postoperative pain (VAS) (6 hours after surgery; postoperative day 1, 2 and 3).  **Secondary outcome**:  Operative time, hemoglobin on postoperative day 1 and 3, complications |
| **Notes** | Korea - Surg Endosc 2011 |

Risk of bias table

| **Bias** | **Authors' judgement** | **Support for judgement** |
| --- | --- | --- |
| Random sequence generation (selection bias) | Low risk |  |
| Allocation concealment (selection bias) | Low risk |  |
| Blinding of participants and personnel (performance bias) | High risk | No blinding, difference in operation techniques |
| Blinding of outcome assessment (detection bias) | High risk | No blinding |
| Incomplete outcome data (attrition bias) | Unclear risk | 4 patients in the LESS group were excluded (no intention to treat analysis) |
| Selective reporting (reporting bias) | Low risk |  |
| Other bias | Low risk |  |

***Kim SM 2015***

| **Methods** | A retrospective study (January 2013 - December 2013) |
| --- | --- |
| **Participants** | 936 women who underwent hysterectomy for benign indication  Setting: unclear, well expereinced gynecology departement in minimally invasive surgery  Inclusion: not defined  Exclusion: suspicion of malignancy or the need for simultaneous interventions such as uterine prolapse repair.  Patient characteristics  Age: TAH 46.3 vs. MPA-TLH 47.7 vs. SPA-TLH 48.8, p=.000  BMI: TAH 25.0 vs MPA-TLH 24.9 vs. SPA-TLH 24.7, p=.582  Uterine weight: TAH 375.8 gram vs. MPA-TLH 258.7 vs. SPA-TLH 262.9, p=.000 |
| **Interventions** | Total abdominal hysterectomy (TAH) (n=284) vs. Multi-port-assisted TLH (MPA-TLH) (n=366) vs. Single-port-assisted TLH (SPA-TLH) (n=286) |
| **Outcomes** | Total blood loss; operative time; length of hospital stay; operative complications |
| **Notes** | Korea - Arch Gynecol Obstet 2015 |

Risk of bias table

| **Bias** | **Authors' judgement** | **Support for judgement** |
| --- | --- | --- |
| Random sequence generation (selection bias) | High risk | Surgical techniques were chosen based on the clinical situation and the surgeon's skill and preference. |
| Allocation concealment (selection bias) | High risk | Retrospective study |
| Blinding of participants and personnel (performance bias) | High risk | Retrospective study |
| Blinding of outcome assessment (detection bias) | High risk | Retrospective study |
| Incomplete outcome data (attrition bias) | Unclear risk | Retrospective study |
| Selective reporting (reporting bias) | Unclear risk | Retrospective study |
| Other bias | Unclear risk | No specific analysis for MPA-TLH vs SPA-TLH, so unclear if patient groups differ. |

***Lee 2015***

| **Methods** | Retrospective cohort study (February 2009 - December 2012) |
| --- | --- |
| **Participants** | 50 patients who had a TLH with or without salpingo-oophorectomy for benign disease of the uterus weighing 500 gram or more  **Setting:** type of hospital not specified, surgeons were defined as well-trained.  Inclusion: -  **Exclusion:** Patients with confirmed malignancies or relatively small uteri weighing less than 500 grams; and patients who received consurrent surgery in additional anatomical areas.  **Patient characteristics**  Age: SPA-TLH 47 vs. Multi-port TLH 46, p=.397  BMI: SPA-TLH 22.6 vs. Multi-port TLH 23.5, p=.560  Uterine weight: SPA-TLH 642 vs. Multi-port TLH 613, p=.200 |
| **Interventions** | Single port assisted TLH (SPA-TLH) (n=25) vs. Multi port TLH (n=25) |
| **Outcomes** | Surgical outcomes (operative time, blood loss)  Hospital stay  Postoperative pain (VAS score at 6; 24 and 72 hours after surgery)  Number of pain killers |
| **Notes** | Korea - Obstetrics & Gynecology Science 2015 |

Risk of bias table

| **Bias** | **Authors' judgement** | **Support for judgement** |
| --- | --- | --- |
| Random sequence generation (selection bias) | High risk | Retrospective study |
| Allocation concealment (selection bias) | High risk | Retrospective study |
| Blinding of participants and personnel (performance bias) | High risk | Retrospective study |
| Blinding of outcome assessment (detection bias) | High risk | Retrospective study |
| Incomplete outcome data (attrition bias) | Unclear risk | Retrospective study |
| Selective reporting (reporting bias) | Unclear risk | Retrospective study |
| Other bias | Low risk |  |

***Li 2012***

| **Methods** | Randomized controlled trial (February 2009 - September 2011) |
| --- | --- |
| **Participants** | 108 patients who underwent laparoscopic hysterectomy for benign uterine disease  **Setting:** Central hospital of Fengxian District Shanghai, surgeries by a single surgical team with the same senior surgeon.  **Inclusion:** Women undergoing laparoscopic hysterectomy; free from a history of heart disease, pneumonia, gastroenteritis and hepatitis, and to have a uterine size equivalent to < 12 gestational weeks.  **Exclusion:** none  **Patient characteristics**  Age: TSPLH 46 vs. TLH 48  BMI: TSPLH 24 vs. TLH 24  Uterine weight: range 158 - 330 gram, no difference (no further data) |
| **Interventions** | Transumblilical single-port LH (TSPLH) (n=52) vs. Traditional four-port TLH (n=56) |
| **Outcomes** | Surgical outcome data (operative time; blood loss and conversion)  Postoperative outcomes  Postoperative analgesia |
| **Notes** | China - The Journal of International Medical Research 2012 |

Risk of bias table

| **Bias** | **Authors' judgement** | **Support for judgement** |
| --- | --- | --- |
| Random sequence generation (selection bias) | High risk | Randomized according to their sequence of admission |
| Allocation concealment (selection bias) | High risk | No concealment |
| Blinding of participants and personnel (performance bias) | Unclear risk | Not reported |
| Blinding of outcome assessment (detection bias) | Unclear risk | Not reported |
| Incomplete outcome data (attrition bias) | Low risk |  |
| Selective reporting (reporting bias) | Low risk |  |
| Other bias | Unclear risk | No baseline pain score assesement (as randomization is inadequate, uncertain if baseline pain scores are well randomized) |

***Wang 2012***

| **Methods** | Prospective cohort compared to retrospective cohort (July 2010 - April 2011) |
| --- | --- |
| **Participants** | 56 patients undergoing hysterectomy  **Setting:** National University Medical Center - all procedures performed by experienced laparoscopic surgeon.  **Inclusion:** an age of 20 years or older, no evidence of gynecological malignancy on imaging studies, an apprpriate medical status for laparoscopic surgery and uterine size of < 400 gram as determined on the preoperative pelvic ultrasound examination. benign or premalignant gynecological conditions  **Exclusion:** uterine size larger than 400 g according to preoperative pelvic ultrasound, suspicion of gynecologic cancer and more than three prior laparotomies.  **Patient characteristics**  Age: single-port group: 46.6 vs. four-port group 48, p=.334  BMI: single-port group: 24.1 vs, four-port group 23.4, p=.443  Uterine weight: single-port: 246.3 vs. four-port group 252.6 |
| **Interventions** | Single-port TLH (n=28) vs. four-port group (n=28) |
| **Outcomes** | Surgical outcomes: operative time; blood loss; change in hemoglobin level  Vaginal stump suture time  Length of hospital stay |
| **Notes** | Korea - European Journal of Obstetrics and Gynecology and Reproductive Biology 2012 |

Risk of bias table

| **Bias** | **Authors' judgement** | **Support for judgement** |
| --- | --- | --- |
| Random sequence generation (selection bias) | High risk | Retrospective data |
| Allocation concealment (selection bias) | High risk | Retrospective data |
| Blinding of participants and personnel (performance bias) | High risk | Retrospective data |
| Blinding of outcome assessment (detection bias) | High risk | Retrospective data |
| Incomplete outcome data (attrition bias) | Unclear risk | Retrospective data |
| Selective reporting (reporting bias) | High risk | Retrospective data + no definition in Material and Methods on complications |
| Other bias | Low risk |  |

***Yim 2010***

| **Methods** | Retrospective cohort study (June 2004 - July 2009) |
| --- | --- |
| **Participants** | 157 patients undergoing laparoscopic hysterectomy  **Setting**: Severance Hospital - Yonsei University College of Medicine in Seoul; 2-surgeon teams (not further specified)  **Inclusion:** Patients undergoing laparoscopic hysterectomy for benign gynecologic conditions and healthy patients (ASA-I and II).  **Exclusion:** Confirmed cervical, uterine or ovarian malignancy; uterine size greater than 16 gestational weeks by pelvic examination; previous history of radiation therapy; and laparoscopic cases that were converted to abdominal hysterectomy.  **Patient characteristics**  Age: SPA-TLH 48.2 vs. C-TLH 48.8, p=.628  BMI: SPA-TLH 23.3 vs, C-TLH 23.0, p=.633  Uterine weightL SPA-TLH 162 vs, C-TLH 123.5, p=.016 |
| **Interventions** | Surgical outcomes (operative time, estimated blood loss, complications)  Resumption of normal diet  Length of hospital stay  Postoperative pain intensity (VAS score) - 6, 24 and 48 hours |
| **Outcomes** | Single port access TLH (n=52) vs. conventional TLH (n=105) |
| **Notes** | Korea - AJOG 2010 |

Risk of bias table

| **Bias** | **Authors' judgement** | **Support for judgement** |
| --- | --- | --- |
| Random sequence generation (selection bias) | High risk |  |
| Allocation concealment (selection bias) | High risk |  |
| Blinding of participants and personnel (performance bias) | High risk |  |
| Blinding of outcome assessment (detection bias) | High risk |  |
| Incomplete outcome data (attrition bias) | Unclear risk | Retrospective study |
| Selective reporting (reporting bias) | Unclear risk | Retrospective study |
| Other bias | High risk | Baseline characteristics significant different for uterine weight and comorbidities |

*Footnotes*

***Chen 2011***

| **Methods** | Randomized Controlled Trial (September 2009 - June 2010) |
| --- | --- |
| **Participants** | 100 patients undergoing a laparoscopic hysterectomy  **Setting:** Tertiary medical center in Northern Tawain, all procedures by a single surgeon, assisted by another surgeon  **Inclusion:** age 30-79 years, ASA score I or II.  **Exclusion:** malignancy, additional adnexal surgery  **Patient characteristics**  Age SP-LAVH 45.7 vs. C-LAVH 48.3, p=.064  BMI SP-LAVH 24.2 vs. C-LAVH 25.0, p=.263  Uterine weight: SP-LAVH 284.9 vs. C-LAVH 227.2, p=.084 |
| **Interventions** | Single-port laparoscopic-assisted vaginal hysterectomy (SP-LAVH) (n=50) vs. Conventional laparoscopic-assisted vaginal hysterectomy (C-LAVH) (n=50) |
| **Outcomes** | Surgical outcomes (Blood loss, operative time, complications)  Time to flatus passage after operation  Postoperative pain score (VAS score) - 12, 24 and 48 hours  Postoperative use of analgesics |
| **Notes** | Taiwan - AJOG 2011 |

Risk of bias table

| **Bias** | **Authors' judgement** | **Support for judgement** |
| --- | --- | --- |
| Random sequence generation (selection bias) | Low risk |  |
| Allocation concealment (selection bias) | Low risk |  |
| Blinding of participants and personnel (performance bias) | High risk |  |
| Blinding of outcome assessment (detection bias) | High risk |  |
| Incomplete outcome data (attrition bias) | Low risk |  |
| Selective reporting (reporting bias) | Unclear risk |  |
| Other bias | Unclear risk | No data on experience of surgeon |

***Choi 2013***

| **Methods** | Retrospective cohort study (April 2010 - April 2012) |
| --- | --- |
| **Participants** | 250 patients undergoing laparoscopic vaginal-assisted hysterectomy  **Setting:** Hospital type not specified, 6 surgeons performed all the cases and surgical techniques were chosen based on the surgeon's skills, preference and clinical situation.  **Inclusion:** benign gynaecologicial diseases  **Exclusion:** malignant diseases were excluded; past abdominopelvic surgery, BMI and uterine size were not considered as exclusion criteria  **Patient characteristics**  Age SPA-LAVH 48.9 vs. MPA-LAVH 48.6, p=.760  BMI SPA-LAVH 24.6 vs. MPA-LAVH 23.5, p=.001  Uterine weight SPA-LAVH 311 vs. MPA-LAVH 339, p=.298 |
| **Interventions** | Single-port access LAVH (n=120) vs. multi-port access LAVH (n=130) |
| **Outcomes** | Surgical outcomes  Complications including conversion |
| **Notes** | Korea - European Journal of Obstetrics and Gynecology and Reproductive Biology 2013 |

Risk of bias table

| **Bias** | **Authors' judgement** | **Support for judgement** |
| --- | --- | --- |
| Random sequence generation (selection bias) | High risk | Retrospective study |
| Allocation concealment (selection bias) | High risk | Retrospective study |
| Blinding of participants and personnel (performance bias) | High risk | Retrospective study |
| Blinding of outcome assessment (detection bias) | High risk | Retrospective study |
| Incomplete outcome data (attrition bias) | Unclear risk | Retrospective study |
| Selective reporting (reporting bias) | Unclear risk | Retrospective study |
| Other bias | High risk | BMI significant verschillende tussen de groepen |

***Eom 2013-1***

| **Methods** | Prospective case-control study (February 2010 - December 2011) |
| --- | --- |
| **Participants** | 399 women undergoing hysterectomy and fulfilled the inclusion/exclusion crieteria  **Setting:** type of hospital not defined, 2 surgeons for conventional LS and 1 surgeon for single port LS performed the cases (defined as expert laparoscopists).Patients chose themselves one procedure  **Inclusion:** women with a uterus a < 16 weeks gestational size on pelvic examination and with a main diagnosis of myoma, adenomyosis, endometrial hyperplasia, or preinvasive cervical neoplasia or women with a cystic adnexal mass < 10 cm in diameter.  **Exclusion:** women with a suspected gynecologic malignancy or endometriosis  **Patient characterisitcs**  Age: SP-LS 40.13 vs, C-LS 41.26, p=.322  BMI SP-LAS 22.77 vs, C-LS 23.44, p=.105  Uterine weight: not specified  All patients received postoperative analgesia via continuous IV infusion. As for additional analgesics, pethidine was used, following that Tramadol or Diclofenac. |
| **Interventions** | Single port laparoscopic surgery (n=116) vs. conventional laparoscopic surgery (n=283)  Speficially: single port LAVH (n=49) vs. conventional LAVH (139) -- but no data for LAVH specific, except pain. |
| **Outcomes** | Postoperative pain (VAS score - 2, 4, 6, 12, 24, 48 and 72 hours) |
| **Notes** | Korea - Journal of laparoendoscopic and advanced surgical techniques 2013 |

Risk of bias table

| **Bias** | **Authors' judgement** | **Support for judgement** |
| --- | --- | --- |
| Random sequence generation (selection bias) | High risk | No randomization |
| Allocation concealment (selection bias) | High risk | No randomization |
| Blinding of participants and personnel (performance bias) | High risk | No blinding |
| Blinding of outcome assessment (detection bias) | High risk | No blinding |
| Incomplete outcome data (attrition bias) | Low risk |  |
| Selective reporting (reporting bias) | Low risk | Only scored for pain |
| Other bias | Unclear risk | Experience of surgeons not further defined than ‘experts’ |

***Hong 2014***

| **Methods** | Computed clinical pathway based case-control study (May 2011 - Augustus 2013) |
| --- | --- |
| **Participants** | 72 patients undergoing laparoscopic hysterectomy  **Setting**: General hospital, not further specified, procedures were performed by a micro-surgery team (2 surgeons). Choice for surgery was made by the patient (and included economic considerations).  **Inclusion:**  **Exclusion**: age < 21 years or > 65 years; moderate-to-servere pelvic adhesions; previous history of radiation or pelvic inflammatory disease; gynecologic malignancy; and allergy to nonsteriodal anti-inflammatory drugs.  **Patient characteristics**  Age: LESS hysterectomy 45 vs. LAVH 45, p=.256  BMI: LESS hysterectomy 25.4 vs. LAVH 26.9, p=.421  Uterine weight: not reported  To compare postoperatively pain objectively, the type and accumulated dose of analgesics used by each patient was calculated and converted to a pain-relief score (computed clinical pathway). |
| **Interventions** | LESS hysterectomy (n=36) vs. LAVH (n=36) |
| **Outcomes** | Operative time; blood loss; complications  Postoperative pain score (VAS) - 2, 24 and 48 hours postoperative |
| **Notes** | Taiwan - Gynecology and Minimally Invasive Therapy 2014 |

Risk of bias table

| **Bias** | **Authors' judgement** | **Support for judgement** |
| --- | --- | --- |
| Random sequence generation (selection bias) | High risk | Patient's choice |
| Allocation concealment (selection bias) | High risk | Patient's choice |
| Blinding of participants and personnel (performance bias) | High risk | No blinding |
| Blinding of outcome assessment (detection bias) | High risk | No blinding |
| Incomplete outcome data (attrition bias) | Unclear risk | Postoperative pain - unclear if missing data |
| Selective reporting (reporting bias) | High risk | Complications not descirbed in the material/method |
| Other bias | Unclear risk | Unclear if LESS was LAVH as well. Never specified. Experience of surgeons not clearly defined. |

***Jung 2011***

| **Methods** | Retrospective cohort study (April 2009 - April 2010) |
| --- | --- |
| **Participants** | 458 patients undergoing laparoscopic hysterectomy  **Setting:** Hospital not specified. One single surgeon performed all the cases.  **Inclusion**: not defined  **Exclusion:** not defined  Patient characteristics  Age S-LAVH 49.76 vs. C-LAVH 48.42, p=NS  BMI S-LAVH 25.39 vs. C-LAVH 24.20, p=NS  Uterine weight: S-LAVH 249.46 vs. C-LAVH 314.99 p<.05 |
| **Interventions** | Singe port LAVH (n=183) vs. conventional LAVH (n=275) |
| **Outcomes** | Operative time; anesthesia time; blood loss  Popstoperative bowel gas passage time  Intra- and postoperative complications |
| **Notes** | Korea - Journal of laparoendoscopic and advanced surgical techniques 2011 |

Risk of bias table

| **Bias** | **Authors' judgement** | **Support for judgement** |
| --- | --- | --- |
| Random sequence generation (selection bias) | High risk | Retrospective study |
| Allocation concealment (selection bias) | High risk | Retrospective study |
| Blinding of participants and personnel (performance bias) | High risk | Retrospective study |
| Blinding of outcome assessment (detection bias) | High risk | Retrospective study |
| Incomplete outcome data (attrition bias) | Unclear risk | Retrospective study  2 patients from the S-LAVH group were excluded after conversion. |
| Selective reporting (reporting bias) | Unclear risk | Retrospective study |
| Other bias | High risk | Groups are comparable  No clear patient characteristics nor indication for surgery defined |

***Kim 2010***

| **Methods** | Prospective (for single port) and retrospective (for conventional) case-control study  (conventional: September 2005 - April 2008; single port: May 2008 - February 2009) |
| --- | --- |
| **Participants** | 86 patients undergoing LAVH  **Setting:** Type of hospital not descirbed, procedures done by single surgeon  **Inclusion:** all consecutive LAVH with symptomatic myomas and/or adenomyosis **Exclusion**: -- **Patient characteristics**  Age: SPA-LAVH 45.9 vs. C-LAVHv 44.8, p=.2  BMI: SPA-LAVH 23.6 vs. C-LAVH 24.9, p=.07  Uterine weight: not specified (only size described in centimeters) |
| **Interventions** | SPA-LAVH (n=43) vs. C-LAVH (n=43) |
| **Outcomes** | Operative time, blood loss, failed cases, transfusion requirements, change in serum hemoglobin, postoperative hospital stay, postoperative pain score (VAS) |
| **Notes** | Korea - Surgical Endoscopy 2010 |

Risk of bias table

| **Bias** | **Authors' judgement** | **Support for judgement** |
| --- | --- | --- |
| Random sequence generation (selection bias) | High risk |  |
| Allocation concealment (selection bias) | High risk |  |
| Blinding of participants and personnel (performance bias) | High risk |  |
| Blinding of outcome assessment (detection bias) | High risk |  |
| Incomplete outcome data (attrition bias) | Unclear risk | Retrospective study |
| Selective reporting (reporting bias) | Unclear risk | Retrospective study |
| Other bias | Unclear risk | Experience of surgeon not described |

***Kim TJ 2015***

| **Methods** | Randomized controlled trial (December 2011 - April 2013) |
| --- | --- |
| **Participants** | 256 patients undergoing hysterectomy **Setting:** eight Korean university hospitals that serve as teaching centers with 14 surgeons with experience in more than 100 multi-port LH procedures and only 5 surgeons had experience in greater than 50 single-port LH at the time of the study. **Inclusion:** elective patients between 20 and 60 years old with symptomatic myomas or adenomyomsis such as dysmenorrhea, pelvic pain or discomfort, urinary symptoms, and a palpable mass of increasing size or menorrhagia for a duration of at least 2 months. **Exclusion:** uterine size of more than 18 gestational weeks by pelvic examination and those at risk of surgical complications due to an underlying medical disease such as heart failure, renal insufficiency, liver cirrhosis, pancreatitis or uncontrolled diabetes. **Patient characteristics** Age: Single port 47 vs. multiport 47, p=.68 BMI: Single port 23.4 vs. multiport 23.4, p=.88 Uterine weight: Single port 280 gram vs. multiport 298, p=.23 |
| **Interventions** | Multiport LH (n=125) vs. Single port LH (n=126) |
| **Outcomes** | **Primary outcomes**: Conversion (defined as the placement of an additional port or conversion to open hysterectomy) and/or complication proportion of the planned procedure **Secondary outcomes**: Postoperative pain (VAS on day 1 and week 1and amount of analgesics taken) and comsetic scar satisfaction (POSAS as measurement tool) |
| **Notes** | Korea - JMIG 2015 |

| **Bias** | **Authors' judgement** | **Support for judgement** |
| --- | --- | --- |
| Random sequence generation (selection bias) | Low risk |  |
| Allocation concealment (selection bias) | Low risk |  |
| Blinding of participants and personnel (performance bias) | High risk |  |
| Blinding of outcome assessment (detection bias) | High risk |  |
| Incomplete outcome data (attrition bias) | Low risk | No blinding |
| Selective reporting (reporting bias) | Low risk | No blinding |
| Other bias | Low risk |  |

***Koyanagi 2011***

| **Methods** | Retrospective study  (conventional: January 2009-June 2009; Single port: June 2009-March 2010) |
| --- | --- |
| **Participants** | 87 patients undergoing LAVH  **Setting:** Type of hospital not descirbed, procedures done by single surgeon  **Inclusion:** all consecutive LAVH, not clearly specified **Exclusion**: -- **Patient characteristics**  Age: SPA-LAVH 45.2 vs. C-LAVH 47.7 p=.08  BMI: SPA-LAVH 22.0 vs. C-LAVH 23.6, p=.03  Uterine weight: SPA-LAVH 324 vs C-LAVH 369, p=.38 |
| **Interventions** | SPA-LAVH (n=50) vs. C-LAVH (n=40) |
| **Outcomes** | Surgical outcomes |
| **Notes** | Japan - Experimental and therapeutic medicine 2011 |

Risk of bias table

| **Bias** | **Authors' judgement** | **Support for judgement** |
| --- | --- | --- |
| Random sequence generation (selection bias) | High risk |  |
| Allocation concealment (selection bias) | High risk |  |
| Blinding of participants and personnel (performance bias) | High risk |  |
| Blinding of outcome assessment (detection bias) | High risk |  |
| Incomplete outcome data (attrition bias) | Low risk |  |
| Selective reporting (reporting bias) | High risk | Excluded converted cases from analyse. |
| Other bias | High risk | Hospital setting, significant difference in BMI, concomittant procedures |

***Lee 2011-1***

| **Methods** | Prospective case-controle study (January 2009 - March 2010) |
| --- | --- |
| **Participants** | 242 women undergoing laparoscopic hysterectomy  **Setting:** University teaching hospital, procedures were done by two surgeons defined as experts for conventional cases and one surgeon for single-port. Patients chose one of the techniques.  **Inclusion**: women with a uterus < 16 week gestational size on pelvic examination; main diagnosis of myoma, adenomyosis, edometrial hyperplasia, carcinoma in situ and microinvasive carcinoma of the uterine cervix; women without suspected uterine or adnexal malignancy or suspected endometriosis and women without uterine descent or a greater than first-degree vaginal prolapse. **Exclusion:** --  **Patient characteristics**  Age S-LAVH 46 vs. C-LAVH 46, p=.723  BMI S-LAVH 25.1 vs. C-LAVH 24.0, p=.347  Uterine weight S-LAVH 246 vs. C-LAVH 256, p=.098 |
| **Interventions** | Single port LAVH (n=80) vs. conventional LAVH (n=162) |
| **Outcomes** | Operative time, hemoglobin changes, return of bowel activity, hospital stay; conversion, complications |
| **Notes** | Korea - European Journal of Obstetrics and Gynecology and Reproductive Biology 2011 |

Risk of bias table

| **Bias** | **Authors' judgement** | **Support for judgement** |
| --- | --- | --- |
| Random sequence generation (selection bias) | High risk | Prospective cohort study |
| Allocation concealment (selection bias) | High risk | Prospective cohort study |
| Blinding of participants and personnel (performance bias) | High risk | No blinding |
| Blinding of outcome assessment (detection bias) | High risk | No blinding |
| Incomplete outcome data (attrition bias) | Low risk |  |
| Selective reporting (reporting bias) | High risk | Inconsistenty in prescribed outcomes in Method section compared to Result section |
| Other bias | Unclear risk | Experience of surgeons not further defined than 'experts |

***Lee 2011-2***

| **Methods** | Prospective case-controle study (January 2009 - December 2009) |
| --- | --- |
| **Participants** | 95 women who are sexually active and underwent LAVH without bilateral salpingo-oophorectomy  **Setting:** University teachting hospital, no information on surgeons. Patients chose their type of surgery  **Inclusion:** premenopausal women who were sexually active; no bilateral salpingo-oophorectomy; indication gynecological benign disease, carcinoma in situ or microinvasive carcinoma of the uterine cervix and no previous treatment inclunding hormonal therapy. **Exclusion:** sexually inactive or menopausal; women who underwent concomitant surgery; women with chronic disease such as diabetes mellitus, hyperention, ischemic heart disease, women diagnosed with known malignancy or endometriosis **Patient characteristics** Age: SP-LAVH 46.40 vs. C-LAVH 45.94, p=.661  BMI: SP-LAVH 25.10 vs. C-LAVH 24.74, p=.660  Uterine weight: SP-LAVH 281 vs. C-LAVH 300.26, p=.577 |
| **Interventions** | SP-LAVH (n=47) vs. conventional LAVH (n=48) |
| **Outcomes** | Evaluation of sexual function (prior and 6 months after surgery with FSFI) |
| **Notes** | Korea - Acta Obstetricia et Gynecologica 2011  Partially same data as Lee 2011-1 |

Risk of bias table

| **Bias** | **Authors' judgement** | **Support for judgement** |
| --- | --- | --- |
| Random sequence generation (selection bias) | High risk |  |
| Allocation concealment (selection bias) | High risk |  |
| Blinding of participants and personnel (performance bias) | High risk |  |
| Blinding of outcome assessment (detection bias) | High risk |  |
| Incomplete outcome data (attrition bias) | Low risk |  |
| Selective reporting (reporting bias) | Low risk |  |
| Other bias | Unclear risk | No report on surgical experience  Groups comparable |

***Park 2015***

| **Methods** | Retrospective cohort study (between 2009 - 2011) |
| --- | --- |
| **Participants** | 1046 women undergoing laparoscopic hysterectomy  **Setting**: type of hospital not reported, type of surgeons not reported  **Inclusion**: patients undergoing LAVH with additional adnexal surgery (salpingectomy, oophorectomy, ovarian or tubal cystectomy and/or cominations of these)  **Exclusion**: other additional procedures (expect specified in the inclusion criteria) **Patient characteristics**  Age: LES LAVH 47.4 vs. C-LAVH 46.6, p=.066  BMI: LESS LAVH 23.85 vs. C-LAVH 23.89, p=.85  Uterine weight: not specified (only height/width and depth reported) |
| **Interventions** | LESS LAVH (n=503) vs. Conventional LAVH (n=543) |
| **Outcomes** | Operative time, blood loss, perioperative hemoglobin level change, transfusion, postoperative hospital stay, perioperative complications (within 30 days), postoperative pain (VAS score, every 8 hours), need for additional analgesics. |
| **Notes** | Korea - Surgical Endoscopy 2015 |

Risk of bias table

| **Bias** | **Authors' judgement** | **Support for judgement** |
| --- | --- | --- |
| Random sequence generation (selection bias) | High risk |  |
| Allocation concealment (selection bias) | High risk |  |
| Blinding of participants and personnel (performance bias) | High risk |  |
| Blinding of outcome assessment (detection bias) | High risk |  |
| Incomplete outcome data (attrition bias) | Unclear risk | Retrospective study |
| Selective reporting (reporting bias) | Unclear risk | Retrospective study |
| Other bias | High risk | Not reported how the selection was made for the type of operation  Groups were not comparable (in LESS group more patients with preovous abdominal surgery and medical co-morbidities)  Type of surgeons not reported |

***Song 2013***

| **Methods** | Randomized controlled trial (January 2010 - January 2011) |
| --- | --- |
| **Participants** | 40 patients undergoing laparoscopic-assisted vaginal hysterectomy  **Setting**: a tertiary teaching hospital, single surgeon performed all the cases (> 500 cases of multi-port and > 200 cases of LESS).  **Inclusion**: indication for hysterecomy, no evidence of malignancy on imaging studies and appropriate medical status (ASA I or II)  **Exclusion:** age <18 years, uterine size > 20 gestational weeks at pelvic examination, recent diagnose of cancer or inability to understand and provide written informed consent.  **Patient characteristics**  Age: LESS 44.6 bd. multi-port LAVH 43.5, p=.42  BMI: LESS 23.2 vs. multi-port LAVH 23.4, p=.86  Uterine weight: LESS 336 gram vs. multi-port LAVH 300, p=.55 |
| **Interventions** | LESS-LAVH (n=20) vs. multi- port LAVH (n=20) |
| **Outcomes** | Primary outcome:  Cosmetic satisfaction (Body Image Questionnaire baseline, 1 week, 4 weeks and 24 weeks)  Secondary outcomes  Posoperative pain (VAS at 12, 24 and 36 hours after surgery, and the amount of analgesics used)  Surgical outcomes (operative time; blood loss; complications) |
| **Notes** | Korea - JMIG 2013 |

Risk of bias table

| **Bias** | **Authors' judgement** | **Support for judgement** |
| --- | --- | --- |
| Random sequence generation (selection bias) | Low risk |  |
| Allocation concealment (selection bias) | Low risk |  |
| Blinding of participants and personnel (performance bias) | High risk | No blinding  CHief reisdent assisting: instered laparosopic ports and sutured the incisions |
| Blinding of outcome assessment (detection bias) | High risk | No blinding |
| Incomplete outcome data (attrition bias) | Low risk |  |
| Selective reporting (reporting bias) | Low risk | Published protocol |
| Other bias | Low risk |  |

*Footnotes*
